# Ten simple rules for avoiding predatory publishing scams

**DOI:** 10.1371/journal.pcbi.1009377

**Published:** 2021-09-23

**Authors:** Michelle Leonard, Suzanne Stapleton, Perry Collins, Terry Kit Selfe, Tara Cataldo

**Affiliations:** 1 Marston Science Library, George A. Smathers Libraries, University of Florida, Gainesville, Florida, United States of America; 2 Digital Partnerships & Strategies, George A. Smathers Libraries, University of Florida, Gainesville, Florida, United States of America; 3 Academic Research Consulting & Services, George A. Smathers Libraries, University of Florida, Gainesville, Florida, United States of America; Dassault Systemes BIOVIA, UNITED STATES

It is easy to imagine predatory publishers ready to pounce at any moment. They present real threats to professional reputations and obstacles on the long road from research to dissemination. It is also easy to be tempted by solicitations for quick and easy publishing, especially for early career faculty and researchers working in high-pressure environments. With a little investigation, though, you can avoid such publishing scams and find the journal that best aligns with your research topic, intended audience, long-term career goals, and complies with any funder’s public access mandates. But what questions should you ask, and what tools are available to help you evaluate whether an invitation to publish, present at conferences, or serve as a peer reviewer is a legitimate opportunity or a trick intentionally designed to dupe you through false flattery? These rules focus on fraudulent publishing but can also be applied to evaluate suspect conferences.

Predatory (also known as problematic, fraudulent, or questionable) publishing covers a complex set of unethical business practices, most focused on extracting money from authors and their institutions. Here, we rely on a definition from the Committee on Publication Ethics (COPE), which describes predatory publishing as “systematic for-profit publication of purportedly scholarly content (in journals and articles, monographs, books, or conference proceedings) in a deceptive or fraudulent way and without any regard for quality assurance” [1]. Fraudulent publishers often misuse models of open access (OA) publishing that charge fees for publication. While this may be controversial, such fees do not define predatory behavior. What sets legitimate publications apart from their problematic counterparts is not prestige, but ethical processes. From review workflows to impact metrics to author fees, predatory journals aim to deceive and exploit.

For science to be credible, integrity of the research and transparency throughout the research life cycle is required. Fraudulent journals and conferences might include valid scholarship, but their abuse of the scholarly process can undermine trust in researchers’ work. Fortunately, tools to tackle the challenge of questionable invitations such as *Think*.*Check*.*Submit*. [2] and *Think*. *Check*. *Attend*. [3] provide guidance on choosing the right path forward for ethical scholarship. These 10 simple rules will guide you through that process.

## Rule 1: Be suspicious

Tread carefully when it comes to unsolicited invitations to submit. How many times a month do you receive an unsolicited email to present at an international conference that covers topics outside of your expertise? Or receive invitations to publish in a journal that you have never heard of? Does the invitation appear professional and legitimate? Do your areas of expertise match the aims and scope of the publication? Is the proposed turnaround time to provide a peer reviewed manuscript reasonable? If you answered “no” to any of these questions, treat them as a yellow flag, warning you to consider whether you are being solicited by a potentially disreputable publisher. Don’t click on provided links that could contain a software virus or lead you to a fraudulent journal website! We recommend you navigate to the journal website directly from your web browser or a reputable database to access the author submission information. Check the URL of the journal and publisher sites carefully, and beware of fake websites that mimic reputable journals.

## Rule 2: Assess the journal content

Once at the journal website, look for signs of credibility. Legitimate publishers provide easy access to the table of contents to their publications. Predatory publishers may not actually publish articles at all, or the quality of published work may be poor. Are current and past issues easily accessed? Are the size and frequency of the issues consistent over the life of the publication? Register a warning flag of concern if you cannot locate or access published issues or their table of contents. Read a few articles in the journal to assess the scientific rigor and whether the contents are relevant to the scope and mission of the journal. When in doubt, grab that cup of coffee and spend time reading what’s inside the cover.

## Rule 3: Check for publication fees (and beware if they are hard to find)

Be sure you understand exactly what you are paying for. Legitimate journals will make any and all fees transparent prior to article submission. Journals that do not post their fees should be viewed with caution. With a growing shift toward OA publishing models that share research without charge to readers, both nonprofit and commercial publishers are experimenting with ways to keep money coming in. One common solution asks authors to pay publication fees known as article processing charges (APCs). These charges may range from hundreds to thousands of dollars per article. It is important to understand that high APCs do not necessarily correlate with fraudulent practice; some legitimate journals charge US$5,000 or more per article. Journals, including both OA and subscription journals, may also incur page charges or fees for value-added services such as translation. Legitimate journals that charge fees for OA publication typically offer waivers for researchers from low- to middle-income countries or who demonstrate financial need. Additionally, large funding agencies typically allow for dissemination costs in project budgets, such as publication fees, in part to support their mandates for public access to research findings. However, not all OA publications assess author fees; the Directory of Open Access Journals lists thousands of OA journals with zero costs to authors.

## Rule 4: Examine the journal’s peer review standards

Peer review is one of the key components to ensuring professional ethics are applied to the process of assessing quality content. Whether the journal employs anonymous review, open review, or another method, check that the review process is well defined. Does the typical duration of peer review match the expectations of rigor? How are biases and conflicts of interest handled? Reputable journals have policies in place to address alleged research misconduct. Reviewers enjoy behind-the-scenes insight into the quality control process for the journal content and give you another view of who finds this journal worthwhile. Check to see if reviewers are acknowledged on the journal website and whether you recognize any of the reviewers as authorities in the field. Today, support is growing for greater transparency in reviews and recognition of reviewers’ credibility and critical service. A number of tools designed to give credit to the traditionally hidden contributions of reviewers are now available such as Clarivate Analytics’ Publons, Elsevier’s Reviewer Hub, and Peer Community In, where reviews may be published or review quality is publicly scored.

## Rule 5: Recognize the gatekeepers

A reputable journal will proudly identify its editors and editorial board members along with their institutional affiliations. Spot-check a few individuals’ professional websites or CVs to confirm their involvement with the journal and to examine if their expertise aligns with the journal’s scope. Be careful not to make assumptions about a journal’s legitimacy based on factors such as editors’ institutional prestige or geographic location or whether or not you know them personally. Lack of inclusive representation among editors is a persistent problem in scholarly publishing, and increased diversity is another sign of a publisher’s commitment to ethical publishing practices.[4] Can you find any description of the editorial process? Editors-in-chief develop dependable editorial boards to share the joy (and burden) of evaluating submissions. Typically, the editor-in-chief conducts an initial assessment to determine if each article fits into the journal scope and mission. And the Chief is where the buck stops; they usually assimilate and deliberate on board members’ recommendations but make the final decision. In between this beginning and end, the Chief’s board members (including section editors and associate editors) solicit appropriate reviewers to evaluate the work and make determinations on innovation, quality, and reproducibility or replicability.

## Rule 6: Discover where the journal is indexed

Consider whether the journal is indexed in reputable and appropriate subject databases. Check the journal’s website for any indexing claims and verify that it is covered by the stated sources. Many databases, such as MEDLINE, Engineering Village, Redalyc, and Web of Science, carefully vet journals for inclusion based on transparent criteria. A quick title search in the database can determine this, or you can check the journal title list to confirm that the journal in question is currently indexed. Another potentially useful resource for identifying the abstracting and indexing sources that cover the journal is Ulrich’s Global Serials Periodicals Directory, which may be available electronically at your library. While inclusion in a reputable database is a good sign of legitimacy, it is not sufficient confirmation of credibility alone; potentially predatory publications have been found in several reputable databases. A journal’s absence from an index does not necessarily mean it is predatory, but a false claim of inclusion, on the other hand, is a clear red flag.

## Rule 7: Verify claims to metrics

Predatory journals don’t shy away from placing fraudulent metrics on their website, like an artificial journal impact factor (JIF). These metrics add an air of legitimacy to their website, but they can also be quick facts you can check to evaluate the publication. For example, if a journal claims a JIF, it must be indexed in the database, Web of Science. If you have access to Web of Science or its companion product, Journal Citation Reports, you can verify the JIF in one of these tools. Wildly popular (and widely criticized [5]) “container level” metrics rank journals by their citation rates (e.g., JIF and SCImago’s Journal & Country Rank) or field normalized metrics (e.g., Elsevier’s Source-Normalized Impact per Paper or Clarivate Analytics’ new Journal Citation Indicator). Some of these research analytic tools are freely available, while others require a subscription. Check with your nearest research university’s library for access.

## Rule 8: Identify the publisher

Look for the publisher’s physical address and personal contact. Can the publisher’s address be verified? If you’re dubious, try to confirm the existence of the publisher in a business registry. In the case of a new journal that doesn’t have an established reputation, knowing the publisher can help establish credibility. If the publisher lists multiple journals on their website, browse their portfolio to see whether there are any titles that you know by reputation. While a lack of other journal titles does not signify anything regarding legitimacy of a publisher, seeing titles of journals you know to be reputable is reassuring. Does the publisher demonstrate adherence to professional publishing ethics through membership in the COPE, International Committee of Medical Journal Editors (ICMJE), the Open Access Scholarly Publishers Association (OASPA), or the Coalition for Diversity and Inclusion in Scholarly Communications (C4DISC)? Predatory publishers engage in unethical business practices with a goal to make money. They make false claims of being a legitimate company and offer promises that are too good to be true.

## Rule 9: Go beyond the lists

We are all busy; we get it. There is too much information out there and not enough time in the day. But ignoring warning signs or shortcutting your evaluation of a potential place to publish your research is not a good idea. Don’t rely on a single, anonymous list to decide this for you. For example, the controversial Beall’s List was taken down in 2017 [6] and has recently been resurrected by one anonymous group and another anonymous person. Where else in scholarly pursuits would you take advice from someone you can’t even name? Cabells is attempting to legitimize their list with more transparency (and a price tag), but the evaluation process is still hard to complete in a one-stop shop. You could use a list as a starting place: “Hmm… someone flagged this journal as predatory at one time, I’m going to go look a little deeper before I submit a manuscript to them.” When you do use a list, be sure you understand the inclusion criteria for a journal or publisher. The scholarly publishing landscape is changing fast. Lists can be outdated or biased. If you find a journal or publisher on a predatory list, don’t write it off until you do some homework. On the flipside, don’t assume a journal is legitimate if it is not on a list. New predatory journals are cropping up all the time. Expect to devote some time to the process of evaluating where you will submit your work.

## Rule 10: Don’t overreact

Don’t let the pressure to publish override your evaluation of the credibility of the publication. The fear of fraudulent publishing might lead authors to dismiss or ignore requests from journals simply because they are new or unfamiliar. Worse, some authors might openly describe such journals as “predatory” to colleagues without investigating the factors above. This does a disservice to legitimate journals that aren’t affiliated with major publishers or may not have published long enough to qualify for coverage in subject indices. It can also be especially harmful to early career journal editors or to journals facing geographic and other systemic inequities in publishing [7]. It’s hard to revive a journal’s reputation once word spreads that it might be predatory. It’s also hard to revive your own reputation if your work is published in a disreputable journal. By considering the rules summarized here and reaching out to journal editors when you have questions or suggestions for improvement, you can avoid falling prey to a scam while encouraging a more inclusive, ethical research ecosystem.

## Conclusion: Ask a librarian

Scholarly publishing is undergoing massive changes, accelerated by digital modes of sharing knowledge. The most important rule to avoid predatory publishing scams is to accept that there is a continuum of legitimacy in publishing venues [8]. Set aside time to investigate where and how you want your work to join the scholarly conversation. Your answer may change over time, with different research outputs, and as scholarly outlets change. When you contribute as an author, confirm the validity of the literature you cite. When you serve as a peer reviewer or editor, verify that you are not promoting predatory publishing. Take advantage of new tools and resources to evaluate credible publishers. Stay vigilant! Predatory publishers are always adapting to try to stay one step ahead with their scams. Check in with a librarian who, as an information professional, can offer useful insights for your quest to find the right outlet for your research.
